# Disinfection of Neonatal Resuscitation Equipment in Resource-Limited Settings: Lessons From a Mixed-Methods Implementation Experience in Kenya

**DOI:** 10.9745/GHSP-D-23-00398

**Published:** 2025-08-14

**Authors:** Anne M. White, Dominic Mutai, Allison Parsons, David Cheruiyot, Beena D. Kamath-Rayne, Joshua K. Schaffzin, Joel E. Mortensen, Amy R.L. Rule

**Affiliations:** a Division of Neonatology, Department of Pediatrics, University of Minnesota College of Medicine, Minneapolis, MN, USA.; b Tenwek Hospital, Bomet, Kenya.; c Rescue: The Behavior Change Agency, Washington, DC, USA.; d American Academy of Pediatrics, Itasca, IL, USA.; e Department of Pediatrics, Faculty of Medicine, University of Ottawa, Ottawa, Canada.; f Division of Infectious Diseases, Immunology, and Allergy, Children’s Hospital of Eastern Ontario, Ottawa, Canada.; g Department of Pathology and Laboratory Medicine, University of Cincinnati College of Medicine, Cincinnati, OH, USA.; h Diagnostic Infectious Diseases Testing Laboratories, Department of Pathology and Laboratory Medicine, Cincinnati Children’s Hospital Medical Center, Cincinnati, OH, USA.; i The Global Collaborating Center for Perinatal Equity, Emory University School of Medicine, Atlanta, GA, USA.; j Divisions of Neonatology and Hospital Medicine, Department of Pediatrics, Emory University School of Medicine, Atlanta, GA, USA.

## Abstract

**Background:**

The majority of neonatal deaths occur in low- and middle-income countries, most often due to perinatal events, prematurity, and/or infection. Reprocessing of neonatal resuscitation equipment is vital for ensuring the availability of clean equipment and preventing transmission of infection to a newborn. Staff at Tenwek Hospital, a tertiary referral hospital in rural Kenya, identified reprocessing medical equipment as a gap in improving neonatal care. We sought to implement steam-based high-level disinfection (HLD) for reprocessing neonatal resuscitation equipment in the labor and delivery ward of Tenwek Hospital.

**Needs Assessment:**

Before implementation, a needs assessment was conducted to identify existing facilitators and barriers to reprocessing through semistructured interviews with key stakeholders at the hospital (N=12) and identify gaps in the hospital’s existing reprocessing procedures. A chemical, chlorine-based method of disinfection was used for neonatal resuscitation equipment in the ward. We conducted baseline bacterial burden of neonatal resuscitation equipment before clinical use, after clinical use, and after reprocessing. There was not a significant decrease in bacterial burden after reprocessing.

**Implementation:**

After implementing a new steam-based HLD process, we conducted bacterial burden testing, which showed a reduction. However, staff preferences and implementation challenges compelled us to modify our original plan and instead implement optimized chemical HLD using chlorine. Although testing showed improved bacterial burden from baseline, in our small number of samples, bacterial burden testing after implementing the optimized chemical HLD process did not differ significantly compared to steam-based HLD.

**Conclusions:**

Optimal chemical HLD was felt to be feasible and sustainable in the local setting. Reprocessing methods should be designed for unique challenges in low-resource settings.

## Abstract (precis)

Infection prevention practices, including the proper disinfection of medical equipment, are key to decreasing neonatal deaths, but in reality, there are many challenges to the implementation of these practices in low-resource settings.

## Teaser

**Key Findings:**

- Local stakeholders at a tertiary referral hospital in rural Kenya identified neonatal sepsis as a significant problem at their facility and decided that disinfection of neonatal resuscitation equipment was the first step in addressing this issue.
- Adaptations were made in the ward to implement a steam-based high-level disinfection (HLD) process, which damaged single-use equipment, so this process was not sustainable at the hospital.
- Pivoting to an optimized chemical HLD based on staff preference and availability of local resources and supply chain realities proved to be a sustainable method of reprocessing. There was no significant difference observed in bacterial counts following the optimized high-level disinfection compared to steam-based high-level disinfection, and both processes were improved compared to the baseline method.

**Key Implication:**

- Implementation of proper disinfection processes must be tailored to local settings and preferences and consider the availability of resources, including clean water, personnel, and space, as well as adequate training and system-wide support for implementation.

## BACKGROUND

Despite global progress in reducing childhood mortality, neonatal mortality rates remain unacceptably high. The burden of neonatal deaths occurs in low- and middle-income countries (LMICs).^1^^–^^3^ The top 3 causes of neonatal mortality are perinatal events (birth asphyxia), prematurity, and infection.^4^^–^^5^ Neonatal sepsis is responsible for nearly 800,000 of an estimated 3 million deaths of newborns around the world every year.^6^ Survivors are at increased risk for comorbidities, such as neurodevelopmental delays, including hearing loss and cerebral palsy.^7^ Studies have demonstrated that LMICs carry a higher burden of neonatal morbidity and mortality compared to high-income countries.^8^^,^^9^ Addressing infections in LMICs is complex and multifaceted, including challenges with prevention, diagnosis, and treatment. Disinfection or reprocessing of medical equipment is an aspect of infection prevention that has recently gained attention.^10^ Eslami et al. described observations of damaged and dysfunctional neonatal resuscitation equipment as well as concerns for potential nosocomial transmission of infection because of a lack of standardized reprocessing.^11^ Historical case reports demonstrate the reality of nosocomial transmission of infection to neonates from contaminated equipment.^12^^,^^13^

In the 1960s, Dr. Earl Spaulding outlined standards for disinfection, describing a system to identify objects to undergo sterilization or disinfection based on the degree of risk involved in their use.^14^^,^^15^ Under this system, reusable medical equipment is classified as critical, semicritical, and noncritical. Neonatal resuscitation equipment, including bag-valve-mask and suction devices, are semicritical items. Accordingly, the minimal level of reprocessing is high-level disinfection (HLD); sterilization is preferred when possible.

In 2016, a group of neonatal experts developed a guideline establishing recommendations for HLD and sterilization of neonatal resuscitation equipment in resource-limited settings based on the U.S. Centers for Disease Control and Prevention and World Health Organization standards for sterilization and disinfection.^16^ It provides step-by-step instructions for 4 primary stages of reprocessing: preparation, pre-disinfection, disinfection, and post-disinfection.^16^ The disinfection stage includes directions to achieve sterilization, the preferred outcome, via autoclave. The disinfection stage also offers 2 primary methods to achieve HLD: heat HLD by boiling or steaming or chemical HLD by chlorine or activated glutaraldehyde.^16^ In preparing the guideline, standard principles were adapted to resource-limited settings based on expert opinion and consensus due to the absence of reprocessing literature from LMICs. However, the guideline relies on several resources that are typically scarce in LMICs, including access to clean water, dedicated personnel for reprocessing with most staff busy managing high-volume/high-acuity clinical environments, and adequate physical space to conduct reprocessing.^10^

The guideline and the issue of reprocessing were felt to be of such importance that it was included in the second edition of the Helping Babies Breathe Program, a highly successful and widely distributed education program designed by the American Academy of Pediatrics in collaboration with other global partners to teach neonatal resuscitation and essential newborn care in resource-limited settings.^17^^–^^20^ Before publication, the efficacy of the guideline was tested in a laboratory in Europe but not in any LMIC settings for which it was designed.^21^

Nurse-midwives and pediatricians at Tenwek Hospital, a tertiary referral hospital in rural Kenya, identified reprocessing medical equipment as a gap in improving neonatal care. They identified the gap through awareness of infections as a significant cause of neonatal morbidity and mortality and recognition of suboptimal infection prevention practices, including reprocessing of equipment. They formed a team with partners from a high-income country (HIC) with a previous history of collaborating with the nurse-midwives, nurses, and pediatricians at Tenwek Hospital to assess the feasibility and then implement the guideline.^22^ We describe the initial evaluation of the reprocessing procedures at the hospital and the experience implementing the recommended reprocessing procedures according to the guideline.

Nurse-midwives and pediatricians at Tenwek Hospital, a tertiary referral hospital in rural Kenya, identified reprocessing medical equipment as a gap in improving neonatal care.

## NEEDS ASSESSMENT

Tenwek Hospital is a tertiary referral center in Bomet, Kenya, with 3,000 deliveries per year and 1,500 admissions to the neonatal unit (including referrals from other facilities). At Tenwek Hospital, nurses and nurse-midwives are responsible for reprocessing equipment in addition to clinical care tasks.

Before beginning implementation of the reprocessing guideline at Tenwek Hospital, we conducted a needs assessment comprised of semistructured interviews with key stakeholders, observations of existing processes at the hospital, and determining the bacterial burden of resuscitation equipment.

### Ethical Approval

Ethical approval was obtained from the Cincinnati Children’s Hospital Institutional Review Board and the Tenwek Hospital Institutional Research Ethics Committee for a multiphase participatory mixed-methods study.

### Interviews With Key Stakeholders

The HIC-based team members conducted individual semistructured interviews with key stakeholders in person and using photovoice, a qualitative research method in which participant-taken photographs and narratives are used to translate experience to actionable knowledge.^23^^,^^24^ Interviews were conducted with 12 maternity and nursery nurses and nurse-midwives and 5 Tenwek hospital administrators to evaluate facilitators and barriers to implementing the new process and to better understand the existing process used for reprocessing equipment.^22^ Issues discussed included their preferences in disinfection methods between autoclave, heat HLD (by boiling or steaming), and chemical HLD.

Interviewees expressed concerns that we categorized into 4 qualitative themes (Table 1): equipment damage, insufficient capacity, process adherence, and effectiveness. Given limited supplies, any process that would damage the equipment raised concerns. Insufficient capacity influenced their preferences between the cleaning options.

**TABLE 1.** Qualitative Themes and Illustrative Quotes From Key Stakeholders Regarding Methods of Disinfection, Tenwek Hospital, Bomet, Kenya

**Theme**	**Process**	**Quote**
Equipment damage	Autoclave	*Autoclave will not work for resuscitation equipment because of the plastic.*
	Boiling	*Worried that boiling over time will make the rubber harder and distort the shape.*
	Chemical	*If we had a way to heat the water, [boiling] would be okay to try because the chemical breaks down the equipment faster.*
	Steaming	*The only thing that can be boiled or steamed is the rubber, not the metal parts.*
Insufficient capacity	Autoclave	*Autoclaving they can put in that machine for 45 minutes. In 45 minutes, I can have 2 deliveries.*
		*If we have enough supply, I will go for autoclave.*
	Boiling	*In our setting, it cannot be like practical because we might not have time unless there is another person who is just in for that.*
		*We need a heater and something to put them in. How much heat would be needed? There’s not a consistent way to determine the heat level. *
	Chemical	*Handling is a problem…sometimes we have an emergency and there is no suction bulb so someone will go and drop it and then pick it and rinse it assuming it is ready.*
	Steaming	*It is more or less the same as boiling. 20 minutes is a long time. You need to locate someone to do that work. *
Process adherence	Overall	*With disinfection there is more temptation for someone to pick it before even the time is over…with the steam there is no way you would pick it before the timer. Also, with the disinfection you have to rinse with sterile water before you use. *
	Autoclave	*So, that’s sort of the issue I see with that. It’s just sort of the cultural thing of waiting til the last minute and autoclaving requires a little bit of thinking ahead.*
	Chemical	*…assuring that it goes through the process is a problem. That’s the big challenge.*
Effectiveness	Autoclave	*On the other hand, we know it works, it’s used for everything.*
	Boiling	*But is it effective?*
	Steaming	*Is it effective?*

*I’m not really in for just only deliveries or just there for admissions. I can also discharge others to go home receiving other complications, even gynecological complication, meeting them, preparing them for…so our time is very limited.* —Interviewee

This provider went on to say that having a person dedicated to the cleaning of equipment would be ideal.

Process adherence concerns did not affect disinfection method preference because, according to interviewees, each method presented unique challenges. Many of those interviewed echoed the sentiment that the biggest challenge was ensuring that the equipment was disinfected.

*Assuring that it goes through the process is a problem. That’s the big challenge.* —Interviewee

As previously mentioned, challenges existed with adherence to reprocessing because of the lack of trained and dedicated reprocessing personnel, with the task falling to providers who were overburdened with many other assigned tasks. Effectiveness concerns shifted preference toward sterilization because interviewees expressed that they knew autoclave worked but were not sure whether the other processes would be as effective. Interviewees stated concerns regarding the equipment’s ability to withstand the autoclave, very limited autoclave capacity at the site, and that it took too much time. Other factors that influenced their method preference included comfort with the existing chemical disinfection process. One interviewee expressed that any process needed to use power and other resources efficiently so that the hospital would not complain. In contrast, a hospital administrator emphasized staff satisfaction.

*I don’t really care what methods you use as long as the nurses are happy, it works, and it doesn’t damage the equipment; as long as it fulfills that criteria.* —Hospital administrator

### Observations of Existing Processes

**FIGURE 1:**
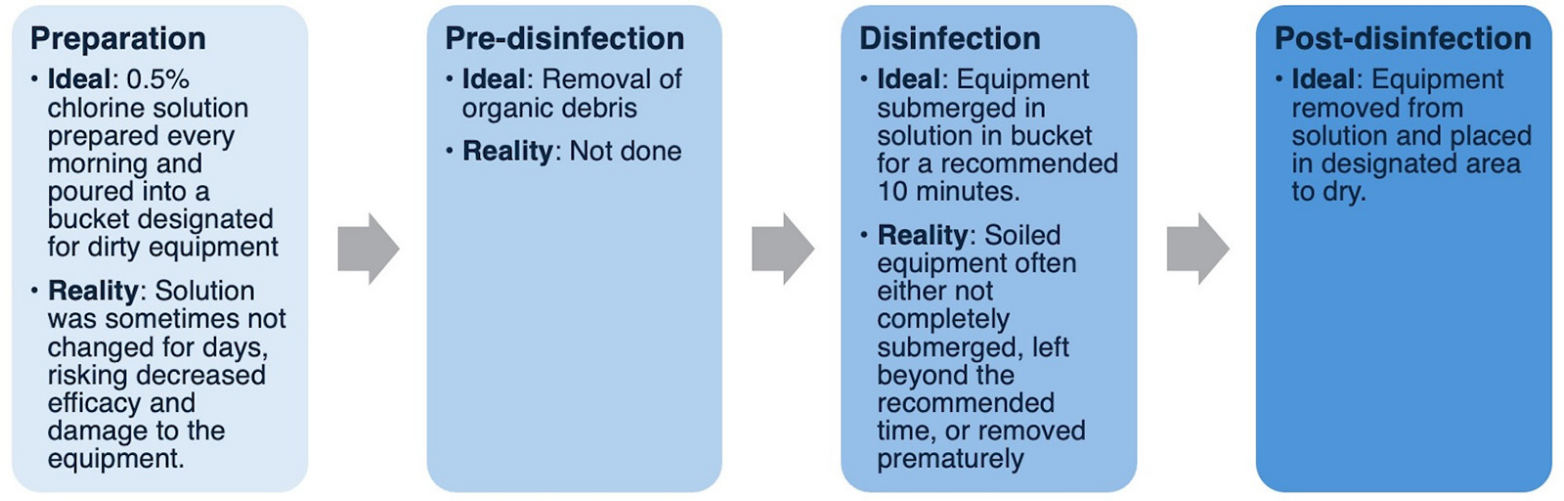
Optimized and Existing Processes Used for Chemical High-Level Disinfection of Neonatal Equipment at Tenwek Hospital, Bomet, Kenya

**FIGURE 2:**
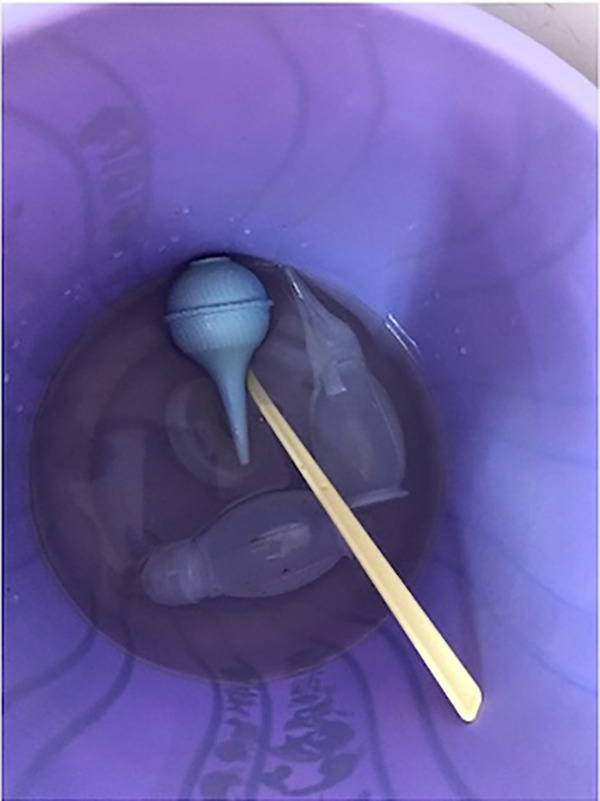
Single-Use Neonatal Suction Bulb, Single-Use Amniohook, Reusable Neonatal Suction Devices, and a Reusable Neonatal Resuscitation Mask Partially Submerged in a Bucket of Disinfectant Solution, Tenwek Hospital, Bomet, Kenya

After the interviews, observations of the existing reprocessing procedures were conducted (Figure 1). Before the recommended processes were implemented, we observed that neonatal resuscitation equipment at Tenwek Hospital was being combined with obstetric equipment to undergo chemical disinfection with chlorine. No pre-disinfection stage was being performed. Pre-disinfection, which is essential for the removal of organic debris (e.g., blood and meconium) that may decrease the efficacy of disinfection, is also important to protect the safety of those handling the contaminated equipment by inactivating viruses (e.g., hepatitis B and C, HIV).^16^ The manufacturer-recommended submersion time is 10 minutes; however, staff observed that soiled equipment was often either left beyond the recommended submersion time or removed prematurely based on equipment availability and clinical needs. The equipment was also often not completely submerged in the chlorine solution as recommended to achieve disinfection, and the solution was only refreshed every few days despite the need to change it every day to maintain efficacy (Figure 2).

Staff observed that soiled equipment was often either left beyond the recommended submersion time or removed prematurely based on equipment availability and clinical needs.

### Baseline Bacterial Burden Determined

**FIGURE 3:**
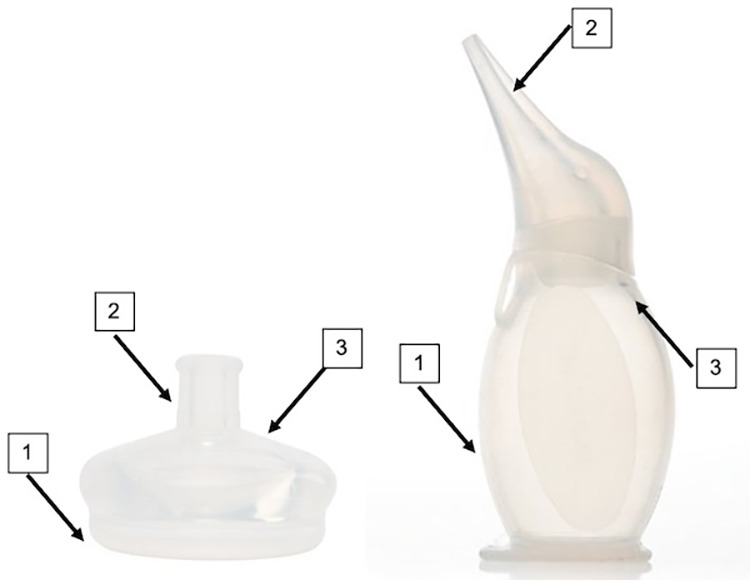
Resuscitation Mask and Bulb Suction With Sample Sites of Testing

At baseline, we determined the bacterial burden of neonatal resuscitation equipment using the existing standard of chemical disinfection semiquantitatively at 3 different time points: before use, after use, and after reprocessing (Figure 3; Table 2).^25^^–^^30^ Cultures of the equipment showed consistent bacterial growth at all time points tested. Notably, there was not a significant decrease in bacterial burden after reprocessing (Table 2).

**TABLE 2.** Baseline Bacterial Burden Data for Select Neonatal Resuscitation Equipment, Tenwek Hospital, Bomet, Kenya

	**Equipment**	**Bacterial Burden, cfu/cm^2^**
Before use	Resuscitation mask	<2.3x10^2^–7.5x10^3^
	Bulb suction	<2.3x10^2^–1.2x10^3^
After use	Resuscitation mask	N/A
	Bulb suction	<2.3x10^2^–8.8x10^3^
After reprocessing	Resuscitation mask	<2.3x10^2^–7.5x10^3^
	Bulb suction	<2.3x10^2^–7.5x10^3^

Abbreviations: cfu/cm^2^, colony-forming units per centimeter squared; N/A, equipment unavailable.

Semicritical equipment should have no culture-detectable microbial growth after reprocessing. Analysis of the data was performed by 2 independent researchers to determine true growth versus contamination. Laboratory work was performed in the microbiology department of Tenwek Hospital. Controls were performed. Laboratory capacity included the ability to perform Gram stain and traditional culture of body fluids (i.e., blood, urine, and cerebrospinal fluid), in addition to some special stains. Culture media, including for our implementation, were prepared onsite by hand; in the case of sheep blood agar plates, the sheep blood was obtained fresh just adjacent to the laboratory. Culture plates were placed into standard incubators following inoculation. A biosafety cabinet was available to handle specimens. One microbiology technologist was assigned to the workspace and performed all routine tasks, including media preparation.

## IMPLEMENTATION

In view of the existing gaps, the stakeholders decided to implement an evidenced-based reprocessing method. Implementation of the new process included 3 phases: (1) training and planning, (2) trial of the new process, and (3) refining and sustaining the new process. For the first 2 weeks of implementation, the team conducted daily meetings with stakeholders to improve the implementation process and discuss challenges and then shifted to monthly meetings to monitor the sustainability of the implementation. A summative narrative of the notes from those meetings is included.

### Phase 1: Training and Planning

In anticipation of the implementation of the steam-based HLD reprocessing guideline, groups of stakeholders gathered at Tenwek Hospital to participate in steam-based HLD trainings. We conducted 3 trainings with groups that consisted of the pediatric and obstetric nurses and nurse-midwives responsible for reprocessing neonatal resuscitation equipment. During these trainings, we simulated steam-based HLD and discussed anticipated barriers to implementing new reprocessing procedures. An anticipated barrier to implementation was the safety risk because of the lack of dedicated reprocessing space. For example, placing a hot pot of water for reprocessing in active patient care areas poses a scalding risk. If the pot was left to boil unmonitored, all the water could evaporate, burning the equipment and creating a fire risk. One suggestion was to build a physical barrier around the pot to prevent it from tipping easily.

Another identified barrier was the inability of staff responsible for reprocessing to consistently follow the appropriate timing of the different steps of disinfection. They cited that the existing process allowed the equipment to sit in disinfectant for minutes to hours, risking either inadequate disinfection or overexposure to chemicals that compromised equipment integrity. There were several questions about putting different types of equipment, such as single-use suction devices and single-use suction catheters, through the process. Although these items are single-use, they are frequently reused in many LMIC settings because of the difficulty in acquiring medical supplies and supply chain inequity.^31^ Because of the lack of data regarding reprocessing single-use equipment, it was unknown if these items would tolerate steam (heat) HLD. The joint decision among stakeholders was to proceed with implementation according to the reprocessing guideline without deviation.

**FIGURE 4:**
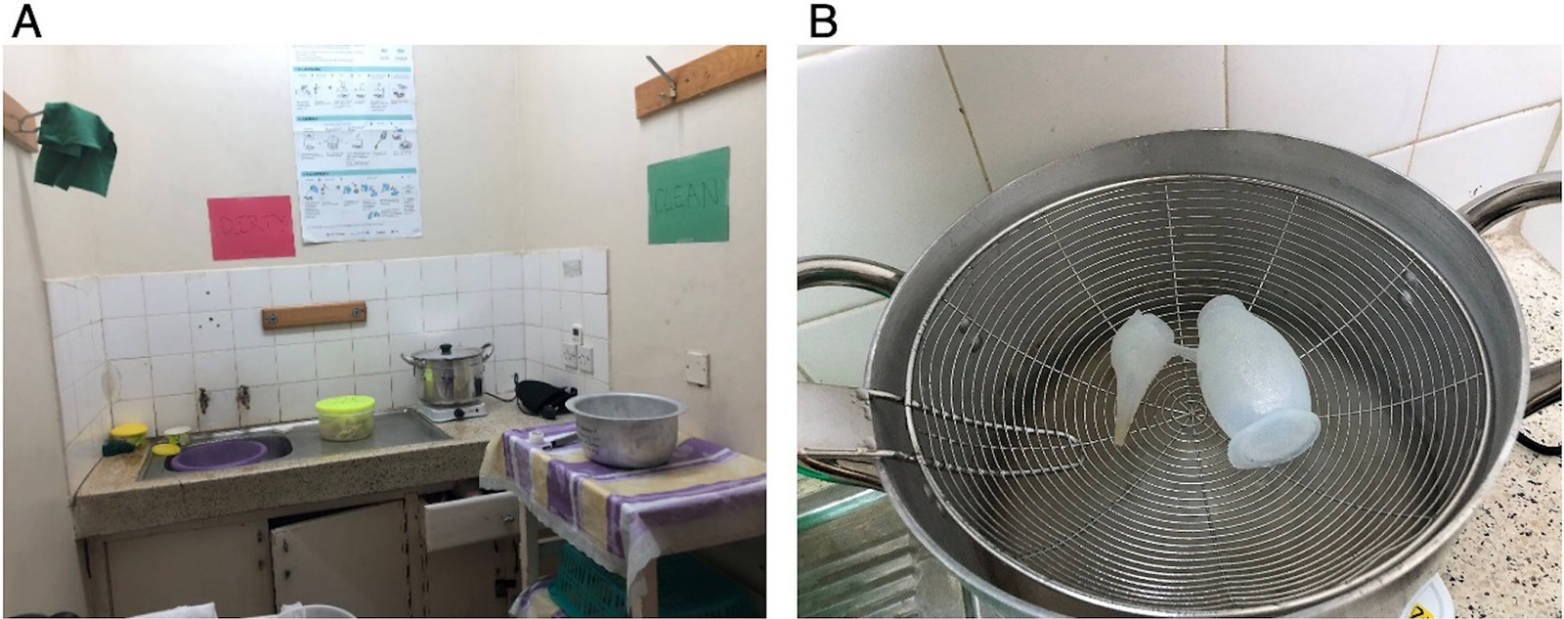
A: Reprocessing Space Created for Steam-Based High-Level Disinfection. B: Reusable Neonatal Suction Device Over an Adapted Steaming Pan, Tenwek Hospital, Bomet, Kenya

Ultimately, a dedicated space for reprocessing was identified in an unused room near the labor and delivery ward (Figure 4). We prepared the space as described in the reprocessing guideline, establishing a dirty-to-clean workflow. The steam-based HLD instructions were posted. Local champions developed a plan to address adherence. Each morning at their handoff, nurses and nurse-midwives decided which team would do the reprocessing for the day. Immediately after handoff, the responsible team (either nurses or nurse-midwives) prepared the reprocessing materials and would be responsible for the reprocessing throughout the day. In Bomet, the town nearest to Tenwek Hospital, we were able to locate all needed steaming supplies except for a steaming pan. In lieu of a specialty steaming pan, we used a shallow strainer along with a standard pot and lid (Figure 4).

During the planning phase, questions arose about whether the available tap water was considered clean and safe to use. Local champions conveyed that the local tap water was filtered on the hospital compound and thought to be clean and safe, but sterile water would be preferred. Although the obstetric department paid for a limited amount of distilled water every day, after it was used for all of the prioritized patient care tasks, there was not enough left to use for reprocessing. Thus, the decision was made to use tap water.

### Phase 2: Trial of the Steam-Based High-Level Disinfection Process

**FIGURE 5:**
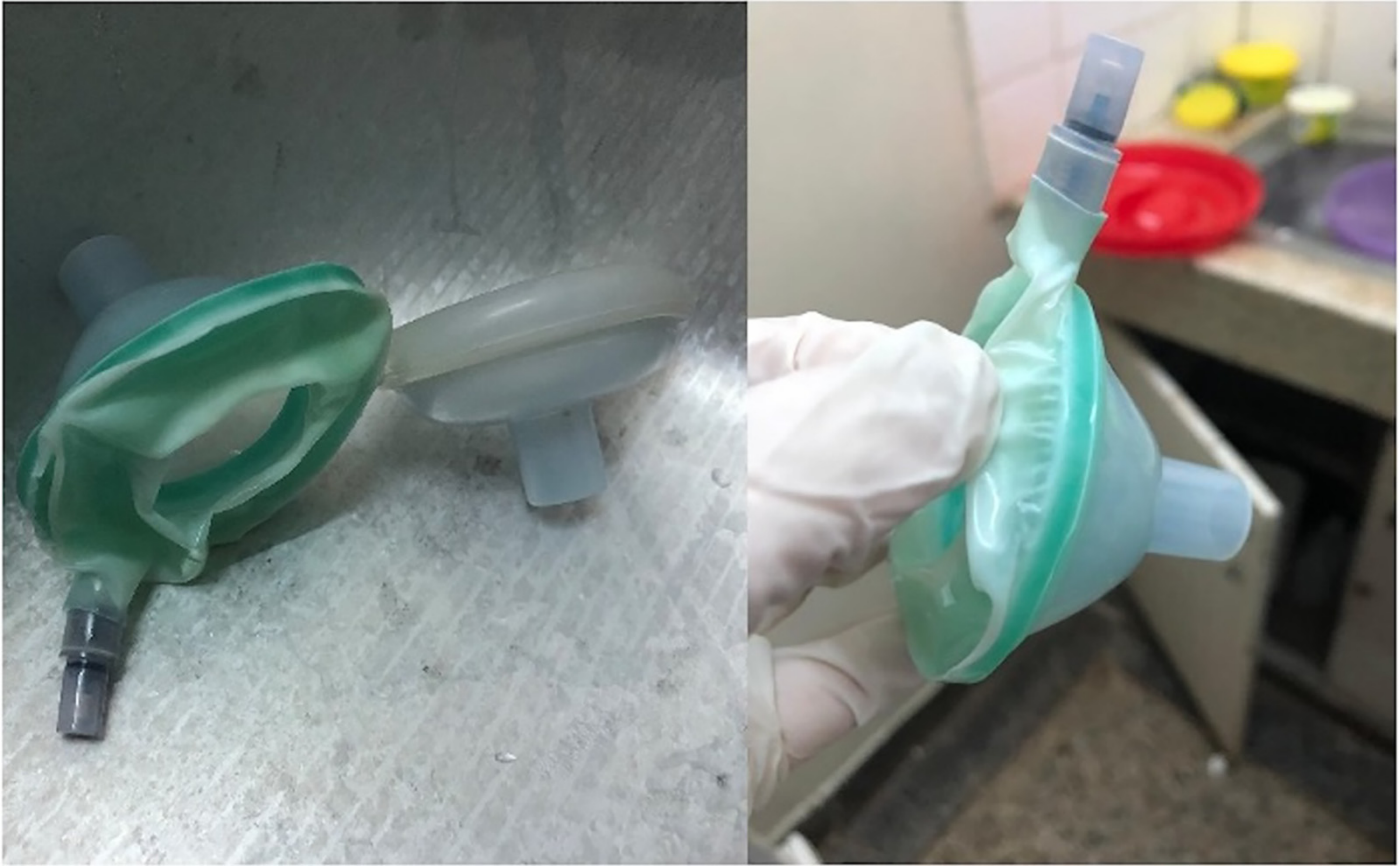
Damaged Masks After Steam-Based High-Level Disinfection

The first items to undergo a trial of steam-based HLD were 2 single-use masks that had recently been used for the resuscitation of neonates. The masks were reprocessed according to the guideline instructions. When the masks were removed after 20 minutes of steaming, they were melted and distorted (Figure 5). These single-use masks were no longer functional and could no longer be used for patient care. Given the reliance of the hospital on single-use equipment as a significant portion of their hospital supply, staff did not feel it sustainable to continue with steam-based HLD. Staff reported it would not be feasible or sustainable to have more than one method of reprocessing in use based on equipment type (e.g., steam-based HLD for reusable and chlorine-based HLD for single-use equipment). Next, we attempted steam-based HLD for reusable equipment for which damage would not be expected. The equipment went through the process unharmed, and bacterial burden was determined before use, after use, and after reprocessing (Table 3). This helped to establish evidence for the effectiveness of steam-based HLD in an LMIC, contingent on the use of reusable equipment. Bacterial testing of equipment after the steam-based HLD process showed a slight decrease in bacterial burden after reprocessing (Table 3).

**TABLE 3.** Bacterial Burden Data of Select Reusable Neonatal Resuscitation Equipment After Implementing Steaming High-Level Disinfection, Tenwek Hospital, Bomet, Kenya

**Time**	**Equipment**	**Bacterial Burden, cfu/cm^2^**
Before use	Resuscitation mask	N/A^a^
	Bulb suction	N/A^a^
After use	Resuscitation mask	<2.3x10^2^–2.4x10^4^
	Bulb suction	<2.3x10^2^–4.1x10^4^
After reprocessing	Resuscitation mask	<2.3x10^2^–7.5x10^3^
	Bulb suction	<2.3x10^2^ –2.9x10^3^

^a^ N/A, not available. Equipment quickly rotated through being used and being disinfected, such that no equipment ever sat too long to be sampled “before use.”

Because steam-based HLD process damaged the single-use equipment that the hospital relies on reusing, this process was not sustainable for reprocessing equipment.

### Phase 3: Refining and Sustaining the New Process

Because of the damage to single-use equipment, steam-based HLD was designated as not sustainable by local providers. As such, we transitioned to a chemical HLD process using the method of chlorine HLD described in the guideline, subsequently referred to as optimized chemical HLD (Figure 1). This optimized method differed from the baseline chemical HLD method with the addition of a pre-disinfection stage to remove visible matter, such as blood or meconium, consistent with the guideline. Before transitioning to the optimized process, we again conducted trainings with the nurses, nursing students, and nurse-midwives responsible for reprocessing.

**FIGURE 6:**
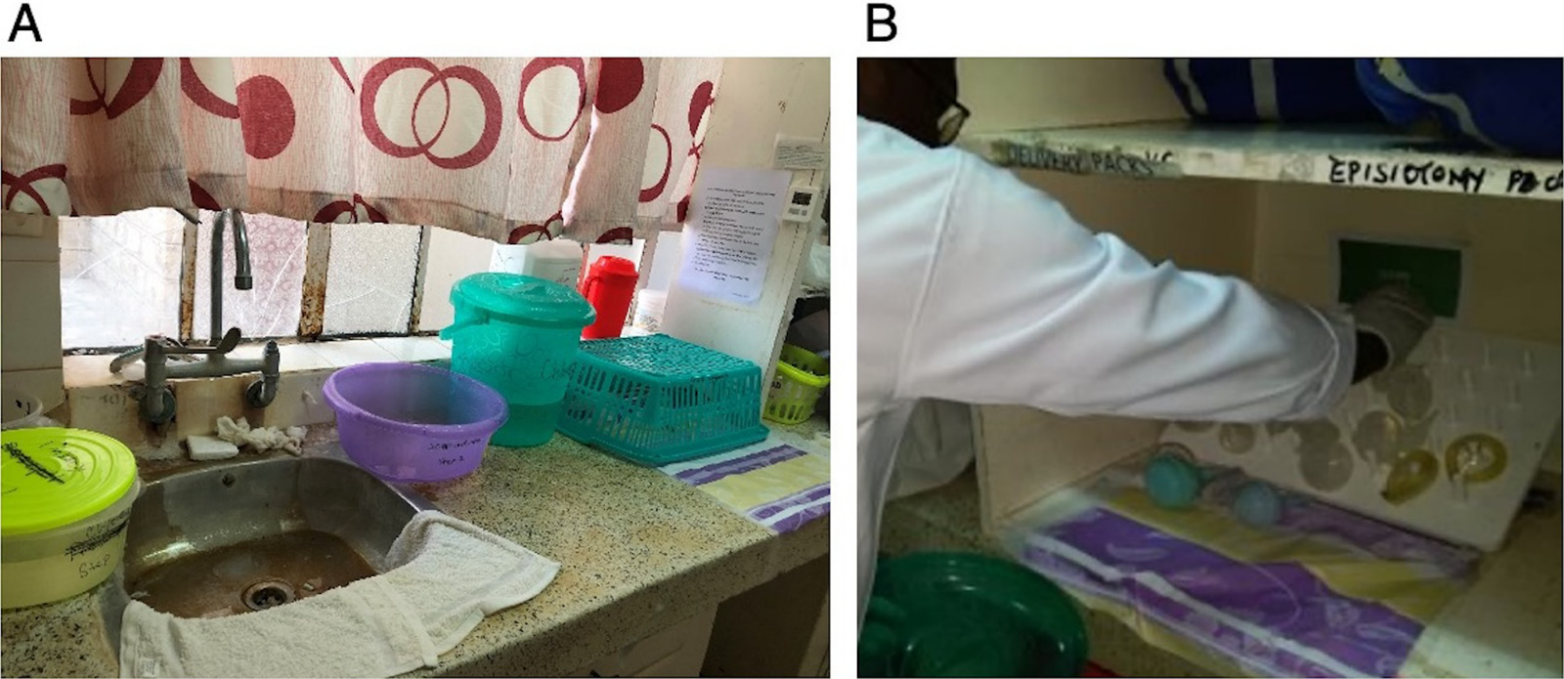
A: Optimized Chemical High-Level Disinfection Reprocessing Space. B: A Midwife Places Newly Disinfected Equipment on a Drying Rack, Tenwek Hospital, Bomet, Kenya

The reprocessing space was relocated to a counter within the labor and delivery ward as the previous space was felt to be inconveniently far away and the need for a heat source was no longer a safety concern (Figure 6). A drying rack, built on request within the hospital compound, created a space to dry and store equipment for immediate use (Figure 6). Reprocessing champions, who were nurse-midwives leading the project locally, were present at the beginning of each shift to conduct trainings and assist with reprocessing of any soiled equipment. We determined the bacterial burden of equipment undergoing this optimized chemical HLD process before use, after use, and after reprocessing. As seen in our data, the optimized chemical HLD process was not inferior to steam-based HLD and did not visibly appear to damage equipment (Table 4). The bacterial burden of equipment after implementing the optimized chlorine HLD process showed a slight decrease after reprocessing (Table 4).

**TABLE 4.** Bacterial Burden Data of Select Neonatal Resuscitation Equipment After Implementing Optimized Chemical High-Level Disinfection, Tenwek Hospital, Bomet, Kenya

**Time**	**Equipment** ^a^	**Bacterial Burden, cfu/cm^2^**
Before use	Bulb suction	N/A^b^
After use	Bulb suction	1.2x10^5^
After reprocessing	Bulb suction	1.8x10^3^

^a^ No masks were available for testing during this time.

^b^ Not available; equipment quickly rotated through being used and being disinfected, such that no equipment ever sat too long to be sampled “before use.”

## LESSONS LEARNED

Reprocessing of neonatal resuscitation equipment is vital for ensuring the availability of clean equipment and preventing transmission of infection to a newborn. A method designed for the unique challenges faced in LMICs is needed. While all documented methods are important and effective, not every method will work for every context.

### Adapt Reprocessing Procedures to Local Context

Steam-based HLD was an effective method of disinfection, but, ultimately, it was not the preferred option for Tenwek Hospital, leading to the first lesson learned during this implementation. Beyond the need for reprocessing to reduce bacterial load, reprocessing methods may need to be adapted to a particular setting in accordance with other balancing measures, such as the availability of local resources (e.g., heat, electricity, water supply, personnel, and supply chain, among others). Within the context of these extenuating factors, an optimized chemical HLD method proved to be the preferred process that could be performed at Tenwek Hospital.

### Continue Training Staff on the Importance of Following Procedures

The introduction of the optimized chemical HLD process has improved the ability of the staff to locate equipment when urgently needed for patient care. The chemical solution is refreshed before each shift to ensure efficacy, and used bulbs, masks, bags, and other delivery room items are cleaned. However, some challenges have also been encountered. Mixing of obstetric and pediatric resuscitation items has been observed. If all neonatal and obstetric equipment goes through each of the reprocessing steps appropriately, this should not be a problem. However, if the process is not followed ideally, there is a risk of maternal microbes contaminating the neonatal equipment, risking neonatal illness. At times, the chlorine solution was not changed as recommended. Whenever the department is busy, impatience with the decontamination process has also been noted. The remedy for these issues has been to sensitize the team on the importance of following the indicated steps to achieve a good outcome. This is done during every ward meeting and at the individual level. All new staff are trained on the chemical disinfection process as a part of their orientation.

### Determine Effective Ways to Reprocess Single-Use Equipment

Another major lesson learned was that reprocessing of single-use equipment is problematic. No guidelines exist for reprocessing single-use equipment because the manufacturers intended for these items to be discarded after one use.^32^ This raises challenging questions about the best way to get supplies of durable equipment to resource-limited settings where reusable equipment may be scarce and reliance on single-use is the only option. In LMICs, single-use equipment comprises a significant amount of the medical equipment cache because of supply chain issues in garnering reusable equipment and a large amount of donated equipment being designed for single use.^31^ More work is needed to understand if there are safe, effective ways to reprocess single-use equipment, especially as supply chain equity interventions will take time. During the COVID-19 pandemic, N-95 respirators, the majority of which are designed to be single-use, were reprocessed.^33^ In addition, delivery of reusable equipment to resource-limited settings needs to be improved—a complex issue beyond the scope of this article but that cannot be ignored.^31^

More work is needed to understand if there are safe, effective ways to reprocess single-use equipment.

### Garner System-Wide Support for Successful Reprocessing Implementation

As a third lesson, system-wide support, including administrative support, is needed to build infrastructure to successfully implement and sustain effective reprocessing. In many LMICs, reprocessing is done by unit clerks, nurses, and nurse-midwives who are responsible for many other duties, including attending to active patient care issues. Reprocessing is done when there is time to do so. When there is no time, reprocessing may be rushed or skipped altogether to have the necessary equipment available for use, resulting in suboptimal disinfection that puts neonates at risk. Hospital systems must recognize the importance of dedicated resources for reprocessing and invest in appropriate training for personnel and adequate space for reprocessing.

Our team also appreciated that one cannot assume or take for granted that there will be clean, running water. At Tenwek Hospital, clean tap water was available through a water filtration unit on the hospital compound. However, in more remote areas, access to clean water is highly variable, which will be an additional barrier to reprocessing.^34^

## IMPLEMENTATION CHALLENGES

Our efforts to validate disinfection methods at the hospital faced challenges in processing and interpreting our microbiology samples. To achieve HLD, a 6-log reduction in bacterial growth should be demonstrated after reprocessing. However, none of the equipment studied during our implementation achieved this. There could be several reasons for this. We worked with small sample sizes. Overcrowded small spaces and a warm, humid environment provided many opportunities for sample and agar contamination to occur. The sheep blood agar needed for bacterial burden testing was made at the hospital using fresh blood from local sheep. While microbiology laboratories in high-income countries frequently receive shipments of ready-to-use media, it is common for laboratories in LMIC settings to make their own.^35^ Even with 2 independent researchers analyzing, our data were difficult to interpret due to a significant amount of background bacterial growth, likely because of both blood agar and environmental contamination. As such, the data are unamenable for formal statistical analysis. This speaks to the challenges of meeting high-income country standards in resource-limited settings, with the need to build laboratory capacity alongside clinical and infrastructure capacity to address neonatal infection gaps.

The baseline data showed that the highest bacterial burden of equipment occurred either after clinical use or after reprocessing. Although growth is expected after clinical use, substantial growth after reprocessing suggests suboptimal disinfection. This is not surprising in this setting. Chlorine is known to be an effective disinfectant but is less potent when diluted with organic materials, such as blood and meconium, both of which are frequently found on used neonatal resuscitation equipment.^36^ Equipment contaminated with blood and/or meconium was observed sitting half-submerged in buckets of disinfectant before implementation. Our baseline data suggest that equipment was being suboptimally reprocessed with inadequate elimination of bacteria; this residually contaminated equipment could be a risk factor for transmission of infection. Data after implementation of both steam-based HLD and optimized chemical HLD showed a slightly decreased bacterial burden of reusable equipment after reprocessing.

### Strengths

This implementation study benefited from an early bidirectional partnership with champions both in the United States and in Kenya. The study was driven by a needs assessment and developed and implemented according to the preferences and resources of Tenwek Hospital. Laboratory capacity at Tenwek Hospital was increased for the implementation with the construction of a new incubator and the local sourcing of sheep blood for the blood agar plates.

### Limitations

The COVID-19 pandemic limited long-term longitudinal follow-up with repeat microbiological testing to assess sustained change over time. Virtual follow-up was conducted, and local champions reported ongoing success with the process. Additionally, there were difficulties analyzing the microbiology data due to a significant amount of environmental contamination. Establishing adequate HLD requires demonstrating a 6-log reduction in bacterial burden. As we were unable to measure with that level of granularity due to contamination and small sample sizes, we instead had to establish that no significant difference existed with the available data.

## CONCLUSIONS

Neonatal infections contribute significantly to global rates of neonatal morbidity and mortality, with the heaviest burden falling on resource-limited settings. Adequate reprocessing of life-saving medical equipment is likely to impact these issues positively. Reprocessing is a complex issue requiring dedicated personnel and space, physical materials, clean water, and an adequate supply of appropriate equipment through supply chain equity. There are several effective methods to achieve adequate disinfection, and reprocessing decisions should be made in conjunction with local champions based on the preferences and resources of each individual site.

## Notes

**Peer Reviewed**
